# The signs of adaptive mutations identified in the chloroplast genome of the algae endosymbiont of Baikal sponge.

**DOI:** 10.12688/f1000research.15841.2

**Published:** 2020-05-14

**Authors:** Sergey Feranchuk, Natalia Belkova, Lubov Chernogor, Ulyana Potapova, Sergei Belikov

**Affiliations:** 1 Limological institute, Siberian Branch of the Russian Academy of Sciences, Irkutsk, 664033, Russian Federation; 2 Department of Informatics , National Research Technical University, Irkutsk, 664074, Russian Federation; 3 Scientific Centre for Family Health and Human Reproduction Problems, Irkutsk, 664033, Russian Federation

**Keywords:** Chlorophyta, Lake Baikal, Chloroplas Genome, Genetic Polymorphism, Mutation Rate

## Abstract

Background: Monitoring and investigating the ecosystem of the great lakes provide a thorough background when forecasting the ecosystem dynamics at a greater scale. Nowadays, changes in the Baikal lake biota require a deeper investigation of their molecular mechanisms. Understanding these mechanisms is especially important, as the endemic Baikal sponge disease may cause a degradation of the littoral ecosystem of the lake. Methods: The chloroplast genome fragment for the algae endosymbiont of the Baikal sponge was assembled from metagenomic sequencing data. The distributions of the polymorphic sites were obtained separately for the genome fragments from healthy, diseased and dead sponge tissues. Results: The distribution of polymorphic sites allows for the detection of the signs of extensive mutations in the chloroplasts isolated from the diseased sponge tissues. Additionally, the comparative analysis of chloroplast genome sequences suggests that the symbiotic algae from Baikal sponge is close to the Choricystis genus of unicellular algae. Conclusions: Mutations observed in the chloroplast genome could be interpreted as signs of rapid adaptation processes in the symbiotic algae. The development of sponge disease is still expanding in Baikal, but an optimistic prognoses regarding a development of the disease is nevertheless considered.

## Introduction

Lake Baikal in South-eastern Siberia is the source of the Angara River and quite close to Baikal – the source of the Lena River; both of these rivers flow far north to the Northern Ocean. The climate in the Baikal region is continental; in winter, the surface of the lake is covered with ice. Many of the plant species in the forests and steppes surrounding Lake Baikal no longer grow anywhere else in the world. Baikal seals, ‘nerpa’, some fish species, and many other creatures that inhabit the lake, adapted to life in the lake, far separated from their closest relatives in the seas and in other lakes.

Baikal sponges live on the bottom of the lake in the coastal zone and have a greenish colour due to their ability to absorb sunlight. The adult sponge *Lubomirskia baicalensis*, after many decades of growth, becomes similar in shape and size to a low branchy tree. The green colour of sponges is caused by a photosynthetic symbiont, a unicellular green alga, adapted to live within the body of sponge cells, contributing in this way to its feeding.

Rapid changes in the ecosystem of Lake Baikal have been observed since 2010–2011. One of the central attributes of these changes is a severe disease and death of sponges, which is now observed in almost all parts of the lake. The symptoms of the disease start with the appearance of pink and brown spots on the surface of the sponge, which eventually lead to complete destruction of sponge tissues. The change in colour of sponge tissue indicates that the chloroplasts of the algal symbiont are damaged from the early stages of the disease.

Changes which happen in the microbial communities of sponges at the beginning of the disease have almost nothing in common, but the most probable cause of sponge disease is arranged and simultaneous attacks of heterotrophic microorganisms of different origin. Common to these pathogens is high changeability, noticed in the relatively high variation of genotypes and in a strategy to be ‘opportunistic’.

A series of research has been conducted on sponges in Baikal and their symbionts, overall signs of crisis in Baikal, and the reasons for sponge disease. Some of the appropriate publications are listed in Table 1, with a comment that the scope of the research is not too wide due to cases of misunderstanding, conflicts of interest and limited funding. But a detailed study of sponge disease provides an opportunity to observe the ways in which the unique ecosystem of Baikal has changed to pass the crisis. Since the advent of molecular biology, no crisis of so large a scale has happened, so the expected results may valuably expand the scope of knowledge about the evolution of alga species.

**Table 1.** References with the scope of research on sponges, their symbionts, ecology and evolution used in the recent study.

The scope of the research	References
Baikal and it's sponges	Chernogor *et al.,* 2013; Pile *et al.,* 1997
Crisis on Baikal	Bormotov, 2012; Khanaev *et al.,* 2018; Kravtsova *et al.,* 2014; Timoshkin *et al.,* 2016
Disease of sponges	Belikov *et al.,* 2019; Feranchuk *et al.,* 2018; Kulakova *et al.,* 2018
Evolution of Algae	Cai *et al.,* 2017; Fucíková *et al.,* 2014; Lemieux *et al.,* 2014; Lemieux *et al.,* 2015; Sun *et al.,* 2016
Adaptation and Evolution	Rosenberg, 2001; Wright, 2004

The algal symbiont of sponge is as conservative as other major constituents of the sponge hologenome in a healthy state. This alga is annotated by taxonomy as a Chlorophyta symbiont of *Lubomirskia* sp. (NCBI Tax. ID 752245); the closest of its relatives is the *Choricystis* genus of coccoid algae. Chloroplasts of the alga are abundant in sponge tissues and are a suitable object of focus for detailed investigations.

The taxonomy of the genus *Choricystis* and other similar unicellular algae from Chlorophyta division has been investigated thoroughly with the use of complete chloroplast genomes. But in a wider context of algal evolution, the credibility of the taxonomy may look insufficient, and advanced methods like taxon sampling, genome synteny analysis or estimates of site heterogeneity have been applied to improve it.

Matters concerning the evolution of a whole clade of algae are anyway ‘enigmatic’. One of the ways to clarify it is to suppose that, in some periods of algal history, the rate of mutations was accelerated for some lineages. The possibility of an accelerated mutation rate, or ‘stress-induced mutagenesis’, or ‘adaptive mutagenesis’, or ‘environment-associated increases in mutations’ is discussed in the theory of evolution but is often treated as controversial. In the case of normal development, a need for accelerated adaptation is compensated by a need to keep the stability of a genotype, and any visible acceleration of mutations is almost excluded. But in the case of the sponge disease, the stress is deadly. And, due to the uniqueness of Baikal, with another look the phenomenon of adaptation may be discovered.

To obtain raw data for the analysis, three samples of sponge tissue were collected and processed using metagenome and metatranscriptome sequencing. This allowed the assembly of a fragment of a reference genome for the alga chloroplast, which covered at least half of the expected length. After this, a straightforward approach to detect a phenomenon of adaptation is to look at distributions of substitutions at polymorphic sites of the chloroplast genome in the three samples.

As one of the precedents of similar studies, the chloroplast genome of an uncultivated alga was obtained from metagenome sequencing data [ Worden *et al.,* 2012]. And, in an extensive study of the population structure of microbial communities [ Truong *et al.,* 2017], distributions of polymorphic sites were carefully investigated. The methods and ideas from those studies were suitable to be applied in the proposed analysis.

A precise taxonomic assignment for the alga species under study is also necessary for consistency of analysis. The composition of alga species across the whole lake is uneven, but the precision of amplicon sequencing only allows the selection of several closest entries in a reference database for the 16S rRNA gene. Classification of the alga species at the level of the 16S rRNA gene has been reported in previous publications about Baikal, and the results from these studies were used as a reference.

## Materials and methods

### Sampling and sequencing

The technology for collection of sponges by scuba divers, with the use of labelled transects, has developed since the detection of sponge disease and is described by Khanaev *et al.* [2018]. The samples collected by divers were immediately placed in containers with Baikal water over ice and transported to the lab, maintaining a constant water temperature.

The three samples of freshwater sponge *L. baicalensis* were collected in June 2016 in the Bol'shiye Koty area (51° 90´ 69 N´´, 105° 07´ 05 E´´) at a depth of 10 m within the same transect. One sample was obtained from a sponge that was healthy in appearance (exhibiting a green colour), one sample was taken from a diseased sponge and one from dead rotten sponge tissues.

Illumina pair-end reads were obtained by DNA metagenome sequencing at Novogene Inc. (Illumina PE 150), for all three samples; in addition, they were processed by a conventional bioinformatics pipeline, which included the filtering of sequencing errors and the assembly of contigs. The extraction and sequencing of RNA samples was also performed at Novogene Inc. This technique was possible only for healthy and diseased sponge tissues; not enough RNA was extracted from the rotten tissues.

### Assembly and annotation of the chloroplast genome fragment

Chloroplast species are abundant enough in the genetic material available from metagenomic sequencing of sponge tissues. A template-based assembly is suitable for obtaining the sequence of a chloroplast genome or at least a substantial fragment of the genome. The sequence of the chloroplast genome of *Choricystis parasitica* (NC_025539) was used as a template for assembly and comparative analysis. This genome is a circular DNA with a length of 94206 base pairs.

To get a refined template from the scaffolds, obtained by de novo assembly of each separate metagenomic sample, scaffolds were selected which had a similarity to the template genome. Then, from cleaned reads, from both DNA and RNA sequencing, reads were selected which had a similarity to any of the selected scaffolds. The reads selected in all samples were merged to a single volume, and both reads in a pair were treated as unpaired. The ‘lightweight’ assembler Inchworm from the TrinityRnaSeq package was then applied to the volume of filtered reads, adjusted to the maximal size of k-mer (K = 31) and minimal sensitivity to sequencing errors. The contigs obtained after the second assembly were compared with the reference genome. This made it possible to select a single contig of 55638 bp in length, which was with confidence identified as a fragment of the chloroplast genome. The stages of the pipeline are listed in Table 2, to explain the data flow at each stage, and to provide the software specifications.

**Table 2.** Stages of assembly and annotation of the chloroplast genome fragment.

	Stage	DNA	RNA	Type of output	Software / reference
1	cleaning	(Outsourced)	Trimmomatic 0.35	reads	Bolger *et al.,* 2014
2	first assembly	(Outsourced) SoapDeNovo	-	scaffolds	Luo *et al.,* 2012
3	template refining	blastn	-	secondary templates	Mature
4	filtering	bowtie 2.2.6		reads	Mature
5	second assembly 2	Inchworm 2.6.5		scaffolds	Grabherr *et al.,* 2011
6	annotation	TrnaSCAN 1.4, Mummer 3.23		genes	Kurtz *et al.,* 2004; Lowe & Eddy, 1997
7	phylogeny	Mafft 7.27, Fastme 2.1.5.1, PhyML 20160207		trees	Guindon & Gascuel, 2003; Katoh & Standley, 2013; Lefort *et al.,* 2015

To check the obtained result, steps 3 and 5 were repeated with other settings: refined templates were obtained by running both blastn with an evalue of 1e-8 instead of 1e-40, and tblastx; the Inchworm assembly was run with default settings instead of the setup with the lowest sensitivity. The assembled contigs were not completely identical to the contig selected as a result, but the difference in any case was fewer than 10 substitutions.

Open reading frames were identified in the obtained contig and most of the identified proteins were annotated following the annotations of the reference genome. TrnaSCAN software was used to identify 13 transport RNA genes in the putative chloroplast sequence, and the locations of 18S rRNA and 23S rRNA were identified by direct alignment with reference rRNAs.

### Identification of polymorphisms

In order to distinguish the polymorphic sites from traces of sequencing errors, their selection in the genome was implemented following a previously published approach [ Truong *et al.,* 2017]. Each RNA and DNA sample was represented as pair-end reads and separately aligned to the assembled fragment of the chloroplast genome using Bowtie2. The alignments were then processed using the Samtools 1.7 software pipeline with conventional settings, and the indexed archives of alignments were used as the input of the algorithm for the identification of polymorphic sites.

When describing the algorithm, *s* represents each position on the alignment of the reads, *N _s_* is defined as the total number of reads and *T _s_* is defined as the number of reads supporting the most abundant allele. Given the sequencing error rate *E*, the non-polymorphic null hypothesis was rejected if the probability that the number of reads equivalent to *N _s_* − *T _s_* coming from the non-dominant allele was < *α* = 0.05. This value was estimated using the probability mass function of a binomial distribution with *N _s_* trials and a successful rate of 1 − *E*. The error rate was set to 0.01 for Illumina sequencing. Bases with a quality below 30 were removed, and reads with an average identity to the reference below 99% were ignored before applying the statistical test. Failing to reject the null hypothesis reflected the absence of alternative alleles or the inability to distinguish between low-coverage potential alternative alleles and sequencing noise.

Therefore, the number of polymorphic sites can be counted for each gene. Another property of each gene is the number of polymorphic sites where the count of alternative alleles is higher than the count of the dominant allele ( *T _s_* < *N _s_* − *T _s_*). This property could be used, in addition to the fraction of mutations in each gene, to detect the intensity of mutations in the DNA and RNA obtained in the samples.

### Phylogenetic analysis

The chloroplast genome sequences of *Picocystis salinarum* (NC_024828), *Myrmecia israelensis* (KM462861), *Botryococcus braunii* (KM462884), *Coccomyxa subellipsoidea* (NC_015084), *Hydrodictyon reticulatum* (NC_034655), *Mychonastes jurisii* (NC_028579) and *Chlorella vulgaris* (NC_001865) were used to reconstruct the phylogenetic tree for the 16S ribosomal RNA (rrs gene) and ATP synthase subunit beta (atpB gene).

The 16S rRNA reference sequences from the Greengenes gg_13_7 database were used to clarify the relations between subspecies of the algae in Lake Baikal. These sequences were identified in the studies of Belikov *et al.* [2019] and Feranchuk *et al.* [2018] as the 16S rRNA templates which are extensively represented in the sponge microbiomes collected in different locations of the lake. Both templates, 4365343 (gb|JF495289.1) and 1118847 (gb|GU936925.1), were directed to the *C. parasitica* rRNA (SAG:17.98, gb|KM462878) as the closest annotated species, by a blast search at NCBI site (98% and 96%).

The nucleotide sequences of the selected genes were aligned using Mafft. A straightforward distance-based approach was used for phylogenetic analysis of the 16S rRNA and atpB genes of the chloroplasts of alga species from the Chlorophyta division. These trees were constructed using Fastme, with a neighbour-joining method to select tree topology and the Jukes–Cantor measure to calculate the distances between genes. A maximum-likelihood algorithm was selected to be used for a tree which was based on 16S rRNA fragments of the *Choricystis* clade, to consider the contribution of the nucleotide substitution model to branch lengths in the constructed trees. A comparison of 16S rRNA genes for the selected strains was performed using PhyML with the default parameters (--model HKY85 -d nt).

## Results

### Remarks about the confidence of the assumptions

Indirect signs of adaptive mutations were detected in the integral distribution of nucleotides in polymorphic sites of the chloroplast genome. The polymorphisms detected in the samples could occur due to sequencing errors or to custom variations in the populations of alga substrains in closely located sponges, not only due to an adaptation to stress.

The samples were collected at the same place, and the alga strain was expected to be the same in all three samples. But micro-populations of algae can anyway be separated in any sponge, and their development can anyway change in response to stress. So, signs of an acceleration of mutations can be demonstrated only at a qualitative level. That is, assuming that there are several substrains of algae in the three samples, which are considered as a single environment, from the distribution of nucleotides at polymorphic sites one can estimate the relative abundance of dominant and alternative substrains in each sample. The observed difference in these abundances can be naturally explained, if the hypothesis about acceleration of mutations is assumed. It can also be explained by another reason, or just accepted as a random case, but to exclude the assumption about adaptive mutations would be a kind of ‘reduction’, an unnecessary simplification of the reality.

This approach should be treated not as a confident explanation of the observed event, but rather as a proof of the possibility. But even an approved possibility of extremely high mutation rates is of value, to clarify the ‘enigmas’ which are noticed in the evolution of algae, and to suggest the ways in which the community of sponges could save itself in a time of crisis.

The questions which should be answered to specify the degree of confidence in the presented results are: how adequate is the assembly of the chloroplast genome? To what extent is the estimated proportion of alternative alleles in polymorphic positions caused by an adaptation, compared with reasons like the natural diversity of genotypes, and with the biases introduced at the experimental and calculation stages?

The fragment of the genome obtained in the assembly is 55683 nucleotides in length, compared to 94206 nucleotides in the circular DNA of the *C. parasitica* chloroplast. An auxiliary run of the assembly pipeline with different settings showed that the fragment considered as the final result does not include at least 5000 nucleotides. But in all cases, the differences were fewer than 10 nucleotides in the whole fragment.

The order of annotated genes in the reference genome corresponded to the order of annotated open reading frames in the assembled fragment, with a difference in a single event of reordering, such that the orientation of the segment from rpoB to tufA genes was reversed. The segment in the reference genome between the rrs and tufA genes was missing in the assembled fragment; instead, the psaB gene from the middle of that segment followed just after the rrs gene. The homology between nucleotide sequences of genes was from 80% up to 98%.

The average proportion of polymorphic positions in all genes was 98.4%, which is comparable with the previously reported 97.8% for bacterial genomes [ Truong *et al.,* 2017]. These values are also consistent with the results reported by Feranchuk *et al.* [2018]. It was shown that on limiting the selection of 16S rRNA fragments of the chloroplast, sequenced by 454 technology, to 95% identity, variation was at most 2% between any of the fragments.

Coverage values for some coding frames were not sufficient to evaluate the distribution properties of alternative alleles. For all the annotated tRNA genes, coverage was at an adequate level, but no polymorphic positions were detected in any of these genes.

However, for 42 of the 56 annotated genes, the coverage was sufficient to detect polymorphisms with adequate significance. Within the purposes of the research, acceleration of mutations may be detected in the distribution of allele frequencies in polymorphisms only at a qualitative level. For qualitative analysis, for each gene and each sample, a coverage of reads was obtained where any of the alternative nucleotides was substituted in a polymorphic position, and those positions were counted for each gene where the coverage of reads with a dominant allele was less than half of the total coverage. These counts were then used to evaluate the abundance of a dominant strain, relative to minor substrains. The fractions of these numbers for each gene are comparable to the fractions for the whole genome, with acceptable dispersion.

The annotation of the alga species under study, with respect to close species of algae, including the species of sponge symbiotic algae in other parts of the lake, was also refined. This made it possible to compare the variations observed between strains in the three samples collected at the same location with those in strains of algae from sponge samples collected in other locations of the lake. The phylogenetic analysis explained above is reported in the last subsection of the results.

### Distribution of polymorphic sites and its interpretation

**Figure 1.**
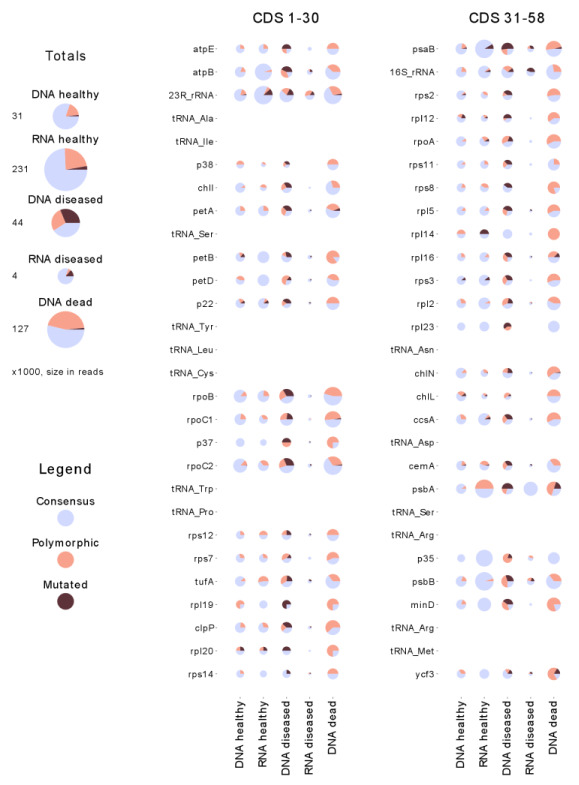
Relative proportion of polymorphic sites in the chloroplast genome of sponge symbionts, for the five samples studied. The results for each of the annotated genes are shown on the right. The left column presents the integrated results for each sample. The proportions of polymorphic sites and sites with high levels of alternative alleles (‘mutations’) are shown as pie charts, relative to the total number of polymorphic sites in all samples. The proportion of sites which are polymorphic in some other samples, but not in the given sample, are shown in light blue. The legend on the left shows the colour scheme used to represent three types of site. The circle radius represents the total number of sequencing reads aligned to the gene segment and used to identify polymorphic sites. The scale of the circle radii is transformed for better appearance, to compensate for the high variation in the number of aligned reads.

The fraction of alternative alleles in polymorphic positions is shown in Figure 1, for a whole set of genes and for each separate gene. Within this fraction, the part is separated where a crucially low (< 50%) abundance of baseline nucleotides was detected in the polymorphic positions.

**Figure 2.**
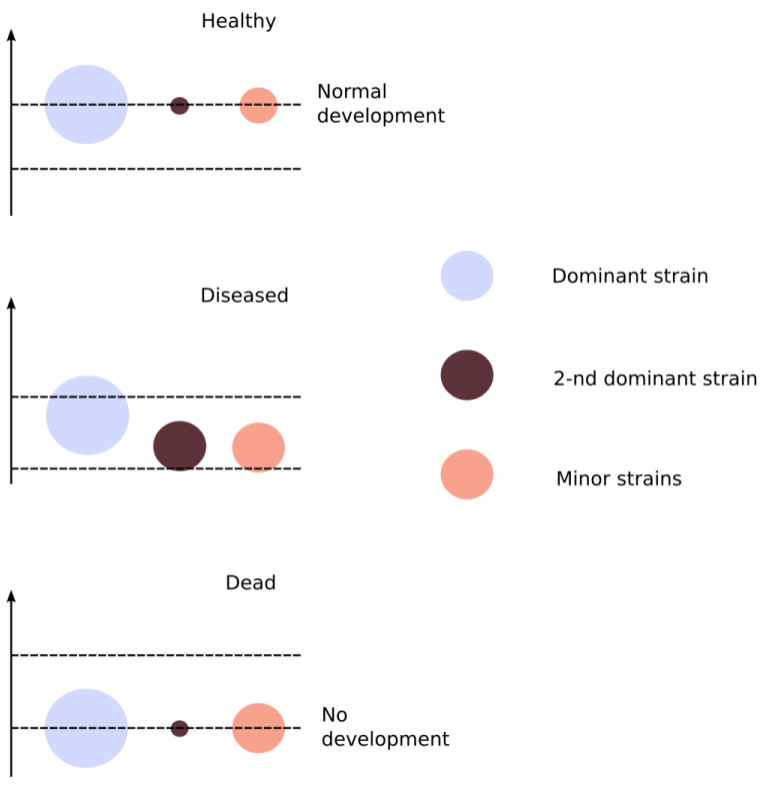
Qualitatively assessed abundance of chloroplast substrains in three samples of sponge. When a sponge is healthy, the major strain dominates, but mutations do not affect the rate of development. This corresponds to a minimal fraction of polymorphisms, and the dominant allele is anyway most frequent in any of the polymorphisms. When a sponge suffers from the disease, it causes, by assumption, an increase in mutation rates and, in turn, an increased abundance and re-distribution of minor strains. This corresponds to an increased fraction of alternative alleles in the genome and, to a lesser extent, in the transcriptome. When a sponge is completely destroyed, just the ‘white noise’ of mutations is found in the genetic material which is left from its chloroplasts.

The separation into three fractions, which is shown in Figure 1, allows one to guess, at a qualitative level, the abundance of the dominant strain, relative to the second most dominant strain and minor strains. Also, comparison of distributions for DNA and RNA allows one to guess how intensive is the development in each of the three separated groups, assuming that RNA synthesis is a sign of any development. This qualitative interpretation is shown in Figure 2.

The precise quantitative estimates of these abundances, to our best knowledge, by no means can be obtained from the available volume of data. The abundance of substrains in the three sponges before the development of disease also cannot be reconstructed.

However, for the sample from the diseased sponge, the fraction of alternative alleles was much higher than in the other two samples. So, as a hypothesis which expands the neutral model, it can be suggested that in the alga cells of a diseased sponge, mutations become accelerated to a rate much higher than any rate compatible with the consistence of metabolic relations. In other words, the living cells in the diseased but alive tissue are desperately trying to survive. Subpopulations of mutated strains develop slowly and die earlier than a dominant lineage – a natural consequence of so many fast mutations in the genome.

But the proposed hypothesis means that the observed acceleration of mutations was a determined event, and some mechanism encoded in the genome triggers this event. But for what reason did this mechanism arise and was it kept conserved in evolution? It is possible just to guess that reason, and several suggestions for that guess are proposed the Discussion section.

### Comparative analysis and taxonomic annotation of symbiotic algae

**Figure 3.**
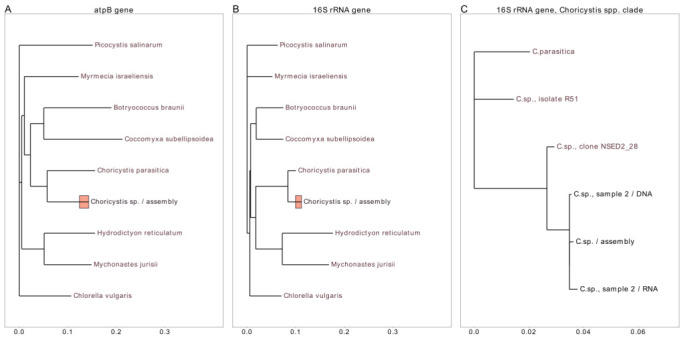
Phylogenetic trees for two chloroplast genes, ATP synthase subunit beta ( **A**) and 16S rRNA ( **B**, **C**). The trees in **A** and **B** are constructed for species within the Chlorophyta division; tree **C** is constructed for species within the genus *Choricystis*. Bars located at the node for the studied chloroplast genome in trees **A** and **B** represent the relative number of polymorphic positions in all five samples, at a 1 : 1 ratio.

The phylogenetic trees for the two selected genes, 16S rRNA and ATP synthase beta ( Figure 3 A, B), in general confirm the conventional relationship between Chlorophyta algae [ Lemieux *et al.,* 2014; Lemieux *et al.,* 2015]. The trees in Figure 3 support the assumption that the symbiotic algae of the sponge *L. baicalensis* are close in taxonomy to the genus *Choricystis*.

The bars in Figure 3 A and B, which show the proportion of polymorphic positions in the genes of symbiotic algae in metagenomic samples, are comparable in size with the scale of distances between genera. The timescale which separates the origins of the close genera in Figure 3 is much larger than the timescale which could adequately interpret the separation of the chloroplast strains detected in the metagenome. So, the observed relation between timescales may be interpreted as being caused by adaptation events, and in that case it can be used to estimate the order of magnitude of the acceleration rate.

Tree C in Figure 3 demonstrates in a more precise way the relation between the species and subspecies of symbiotic algae. The tree is composed an rrs gene for the reference species of *C. parasitica*, the two abundant chloroplast rRNA templates for the sponges from other parts of the lake, and three consensus sequences for the three sponges under study. From these three sequences, one represents the 16S rRNA gene from the reference assembly, and two sequences represent specifically the sample of diseased sponge, as a DNA and as an RNA. The consensus sequences for the latter two datasets were created from the distribution of polymorphic positions.

The chart in Figure 3C illustrates in more detail the estimates of acceleration value provided below. The following assumptions can be introduced: the fraction of polymorphic positions in the whole chloroplast genome is 1.6%; the age of Baikal is about 20 million years, but let the separation of the alga species in Baikal from the rest of the *Choricystis* lineage be about 1 million years ago, and the genetic distance from the alga species under study to *C. parasitica* be about 20%; the fraction of mutations in the diseased sample is assumed to be 12.5% in polymorphic positions and 0.2% in the whole genome; the disease was developed in one season.

Under these assumptions, the relative increase of mutation rate in diseased sponge is estimated to be 10000. Such high acceleration is obviously incompatible with life but, as a comparable precedent, the mutations in cells of some tumours are accelerated 200 times [ Bielas *et al.,* 2006] and even up to 10000 times [ Berger *et al.,* 2012].

## Discussion

The suggested proposal, that the acceleration of mutations in diseased sponge is a cause of the difference observed between distributions at polymorphic sites, is a too simplified model for the phenomenon observed. But, if a huge acceleration of mutations did anyway take place in a determined response to the disease, what could explain the presence of this determinism, if such an acceleration will inevitably lead to death?

A sponge is an organism which should be considered, to a greater extent than other groups, in the context of its symbionts, as a part of a hologenome. A response to attacks of pathogenic microbes in that meta-organism is, mainly, a load to the symbionts but not to the sponge itself. The strategy of most pathogens is to switch to a phase of aggression, after they adapt in a hologenome as a symbiont. And the decrease of biodiversity in healthy sponges, in comparison with times before the crisis [ Belikov *et al.,* 2019], is evidence that the system of discrimination between friends and foes is malfunctioning so that the sponge rejects some of its allies which were constituents of its healthy conservative hologenome.

The question is, how can a sponge survive the crisis, even in a hypothetical case? The only direction is to strengthen the connections within the healthy part of the hologenome, excluding in this way the need for symbiosis with external microbes which may turn out to be opportunistic pathogens. But this require a synchronous adaptation of all constituents of the healthy hologenome, with rates as high in the short period of crisis as when the sponges in Baikal were developed as species.

Unusually high rates of mutations, which were, by assumption, observed in the chloroplast of photosynthetic algae, would in most cases lead to the death of cells with a modified genome, and this will happen a bit earlier than the death of cells with an unmodified genome. And the cause of inhibition of these cells is mostly mutations in genes which are responsible for metabolic relations with other species. But, as was mentioned above, the stage of accelerated mutations cannot be avoided in a way to allow the survival of the whole hologenome and for the algae as its constituent. However, survival is possible if synchronous mutations result not in suffering but in strengthening of the metabolic relations between constituents of the hologenome.

The probability of the proposed scenario is extremely low. But, at least, the hypothetical possibility of this scenario provides a trade-off and balance to be considered in the developmental strategy, instead of inevitable death. The expectations of this scenario can explain the presence of determinism in the initiation of increased growth of the mutation rate. The chance of the species surviving in this scenario is extremely low. But the assumption that similar episodes happened anyway in previous stages of algal evolution would provide an additional degree of freedom, sufficient to explain many of the ‘enigmas’ in the history of algal development. Although the exact periods of these crises are unlikely to be reconstructed, the survival of ancestors of modern algae in these crises could explain a need to keep in their genome a way to trigger again the accelerated adaptation.

## Data and software availability

The nucleotide sequence of the chloroplast genome fragment is deposited to GenBank (ID: MH591948).

The reference project IDs for the nucleotide archives used in the study are PRJEB281624 (metagenomic and metatranscriptomic sequencing) and PRJNA369024 (16S rRNA gene sequencing).

The sequencing reads and source codes of scripts sufficient to reproduce the presented results are available at GitHub: https://github.com/sferanchuk/bsponge_chloroplast.

Archived source code at the time of publication is available at: https://doi.org/10.5281/zenodo.1326765. License: CC BY 4.0 [ Feranchuk, 2018].

Custom scripts on Python (v 2.7) were used to run the pipeline and present the results. The Python libraries pysam (0.14.1), biopython (1.66) and matplotlib (2.2.2) are required to run the scripts.
