# Supplementary material for: The signs of adaptive mutations identified in the chloroplast genome of the algae endosymbiont of Baikal sponge
Source: F1000Res. 2020 May 14;7:1405. Originally published 2018 Sep 4. [Version 2] doi: 10.12688/f1000research.15841.2 (PMC7670478; doi:10.12688/f1000research.15841.2)
Supplement: Peer review history

# Peer review: Reviewer response for version 2

**DOI:** 10.5256/f1000research.25657.r74617

**Published:** 2020-11-23

**Authors:** OPhir Nave

**Affiliations:** 1 Department of Mathematics and Computer Science, Jerusalem College of Technology, Jerusalem, Israel

The paper is very interesting and has an important application. 

- The abstract summarized the main results of the paper. 
- The authors revised the introduction according to the comments. 
- **The introduction is not complete. The authors must add a relevant list of references to this section.** 
- 5 paragraphs of the introduction are without even one reference.
- Also the section "Materials and methods" is without an extensive list of references. This section is very important, and the authors need to present the relevant research list.  
- The results section needs to be revised. Since as it is present in the current version, it is not absolutely clear what the authors achieve from the study. 
- The analysis of the FIgures must be ended in detail. 

Is the work clearly and accurately presented and does it cite the current literature?

No

If applicable, is the statistical analysis and its interpretation appropriate?

I cannot comment. A qualified statistician is required.

Are all the source data underlying the results available to ensure full reproducibility?

Yes

Is the study design appropriate and is the work technically sound?

No

Are the conclusions drawn adequately supported by the results?

No

Are sufficient details of methods and analysis provided to allow replication by others?

No

Reviewer Expertise:

Mathematical model and application to engineering and medicine

I confirm that I have read this submission and believe that I have an appropriate level of expertise to confirm that it is of an acceptable scientific standard.

# Peer review: Reviewer response for version 2

**DOI:** 10.5256/f1000research.25657.r74181

**Published:** 2020-11-16

**Authors:** Ashraf Al-Ashhab

**Affiliations:** 1 Microbial Metagenomics Division, Dead Sea and Arava Science Center, Masada, Israel

The authors have based their study on chloroplast polymorphic sites in algae endosymbiont of Baikal sponge of diseased, healthy and dead sponge tissue. 

- The main concern is that they based all their analysis and conclusions from one sample following each category. The authors also used 150 PE sequencing, while for better assembly and more accurate evaluation of polymorphic sites they could have used a longer amplicon sequencing. 
- The authors have used PE reads to obtain while in the analysis they were treated and independent unpaired read, why did they do this? At which stage the sequencing errors were checked and quality control used is not clear. 
- The authors have used a genome of *Choricystis parasitica* (NC_025539) as a template assembly, and not enough information of why they have chosen this and not others and more than one. 
- Methods on RNA and DNA extraction, library preparation, and size selection are not listed in the manuscript.
- The authors also should indicate some information about contigs length distribution following assembly.

Is the work clearly and accurately presented and does it cite the current literature?

Yes

If applicable, is the statistical analysis and its interpretation appropriate?

Partly

Are all the source data underlying the results available to ensure full reproducibility?

Partly

Is the study design appropriate and is the work technically sound?

Partly

Are the conclusions drawn adequately supported by the results?

Partly

Are sufficient details of methods and analysis provided to allow replication by others?

Partly

Reviewer Expertise:

microbiome

I confirm that I have read this submission and believe that I have an appropriate level of expertise to confirm that it is of an acceptable scientific standard.

# Response

**Published:** 2020-11-16

**Authors:** Sergey Feranchuk

**Affiliations:** Unaffiliated, Russian Federation

Many thanks for the approving.

 I agree and confirm that the study has been done with methodological inconsistencies which are mentioned in the concerns. A consistent research should be based on several replicates of sponges in each phase of disease; sequencing should be more fine-tuned, more deep and should make use of long amplicon extraction. The answers are in the reality of scientific business rather than in the science itself so I would like to omit the more detailed explanations.

 Answering the more specific concerns:

 - Reads were unpaired and mixed after filtering on a template of a similar chloroplast. After this stage, the chances of mis-assembly which are intended to be reduced by a filtering of mate-pairs, were of lesser importance than a need to obtain as long contig as possible, from a filtered pool of reads.

 - A close similarity of the target algae species to *Choricystis* species was observed earlier in vitro [Chernogor 2013] and by 16S rRNA annotation to Silva database.

 - In the primary Inchworm assembly, the targeted 55K contig was followed just by "noisy" contigs with lengths not more than 5K b.p., so the distribution of contig lengths was not explicitly indicated.

# Peer review: Reviewer response for version 2

**DOI:** 10.5256/f1000research.25657.r63440

**Published:** 2020-05-28

**Authors:** Roman Kondratov

**Affiliations:** 1 Center for Gene Regulation in Health and Disease (GRHD), Cleveland State University, Cleveland, OH, USA

The authors addressed my concerns. I am fine with the current version.

Is the work clearly and accurately presented and does it cite the current literature?

Yes

If applicable, is the statistical analysis and its interpretation appropriate?

Not applicable

Are all the source data underlying the results available to ensure full reproducibility?

Yes

Is the study design appropriate and is the work technically sound?

Yes

Are the conclusions drawn adequately supported by the results?

Partly

Are sufficient details of methods and analysis provided to allow replication by others?

Yes

Reviewer Expertise:

NA

I confirm that I have read this submission and believe that I have an appropriate level of expertise to confirm that it is of an acceptable scientific standard.

# Peer review: Reviewer response for version 1

**DOI:** 10.5256/f1000research.17291.r42769

**Published:** 2019-01-23

**Authors:** Roman Kondratov

**Affiliations:** 1 Center for Gene Regulation in Health and Disease (GRHD), Cleveland State University, Cleveland, OH, USA

The massive presence of sponges is known as playing a leading role in the process of biofiltration of Baikal's water. Recently the cases of sponge disease have been expanded rapidly. The significance of the current study is coming from the attempt to investigate molecular aspects of sponge disease using a systems biology approach. In the present submission the authors provide data on the genome and transcriptome of chloroplasts from symbiotic algae. Metagenomic and metatranscriptomic sequencing data was used for a template-based assembly of the chloroplast genome of algae, and a mapping of sequencing reads to the obtained genome sequence was a subject of interpretation, based on a detection of polymorphic sites. The advantage of the study is the exploration of natural samples, improvement of methods of bioinformatical analysis and provocative ideas. I think that the manuscript deserves to be indexed, but there are concerns which the authors need to address: 

1. What was the number of analyzed biological replicas or independent samples? Do the authors expect the same spectra if another sample(s) of sponge tissues would be processed with the same pipeline? What would be the distribution of variations in polymorphic sites in another series of experiments?
2. Were the “healthy”, “diseased” and “dead” samples collected from the same spot? How can the authors be sure that they are dealing with the same species? Could it be that what the authors proposed as “adaptive mutations” is in fact a difference between different subspecies?

I will recommend to tone down the conclusions and to discuss possible alternative interpretations. The statement on a presence of adaptive mutations in the chloroplast genome is too strong. It requires confirmation with more independently obtained samples. There is also no evidence that even if these mutations will be confirmed, that they are “true adaptive” mutations and provide the organism with any physiological advantage. Therefore the title is misleading in my opinion.

 I suggest also to provide more details of the overall design of the study and on novelty in bioinformatics approaches.

Is the work clearly and accurately presented and does it cite the current literature?

Yes

If applicable, is the statistical analysis and its interpretation appropriate?

Not applicable

Are all the source data underlying the results available to ensure full reproducibility?

Yes

Is the study design appropriate and is the work technically sound?

Yes

Are the conclusions drawn adequately supported by the results?

Partly

Are sufficient details of methods and analysis provided to allow replication by others?

Yes

Reviewer Expertise:

Gene expression, biological rhythms, metabolism

I confirm that I have read this submission and believe that I have an appropriate level of expertise to confirm that it is of an acceptable scientific standard, however I have significant reservations, as outlined above.

# Response

**Published:** 2020-04-04

**Authors:** Sergey Feranchuk

**Affiliations:** Unaffiliated, Russian Federation

Many thanks to Prof. Kondratov that he had found a possibility to read carefully the manuscript, so that he had found deep and precise issues in which where the proposed results can be doubted. I did consider his remarks in a most full extent.

I respect the experience of Prof. Kondratov who did suggest a presence of several subspecies of algae in a sponge host. In the revised version I explicitly told about possible presence of subspecies in the samples and add an extra chart which shows the distribution of algae subspecies around Baikal. And I did explicitly specify some of most important details of the study, and did remove some extra material from the second edition, trying to keep the format and to put more accents on the main of the declared results.

I did change the declared statement of the manuscript, to propose a proof of a possibility rather than a proof of an observation. In the revised version I tried to provide a more explicit "proof of possibility", that the adaptation can be just one of the many contributions to the observed distribution of polymorphic sites. And I add an additional argumentation why just a possibility of the adaptation is worth to be presented as a scientific result. In short, the situation around Baikal is complicated, and just an idea how this complication can be resolved is worth to be said.

# Peer review: Reviewer response for version 1

**DOI:** 10.5256/f1000research.17291.r42738

**Published:** 2019-01-08

**Authors:** Michael G. Sadovsky

**Affiliations:** 1 Siberian Federal University, Krasnoyarsk, Russian Federation

Well, on one hand I should say ``Yes’’ to this submission. At least, it completely meets all the up-to-date customs and observances in genomics and molecular biology. On the other hand, the authors rely on a number of (quite complex and apparent) software packages, workbenches and pipelines, and this is the matter of my general scepticism. Actually, we all become the hostages of those software tools; one has to trust software designers and believe the output of the programs is correct, free of mistakes, stably working, etc. Somebody may say ``You, physicists and mathematicians, do use Wolfram’s *Mathematica* and there is no collapse in math, nor in physics’’. Reciprocally, I would like to draw attention to an Elsevier journal devoted to Microsoft Excel errors solely (McCullough and Heiser, 2007 ^1^).

 I am far from the idea to say that the study is wrongly arranged or badly accomplished; I just want to stress the point that there should be some special efforts done to ensure the results are at least stable. For example, what happens with assembled contigs, if we randomly remove a small part of reads? If a series of runs of an assembler yields (almost) the same contigs set, then the results could be used for further analysis. The problem arises, if one gets a number of sets of contigs with a sounding difference between them. The paper has no answer on that point; a comparative study (like that one presented by the authors) should have some proofs of the absence of artefacts affecting the comparison of fine differences between biological objects involved in the study. Meanwhile, I pretty well understand that such output testing falls beyond the customs and habits of NGS sequenced data treatment and I am in the smallest minority. So, from that point of view the paper completely meets all the custom data treatment procedures and in such capacity should be recommended for indexing.

 Another important issue of the paper is that it presents an attempt to tie together ecological (environmental) processes, and some genetic background that may stand behind. Here the word `crisis’ used by the authors makes a point: regularly, *ecological crisis* is stipulated as a rather fast running process in a community resulting in serious (and inevitable) loss of the greater part of species from the community. Maybe, this word is too strong here: what if the observed infection intrusion is just a regular (while long ranged) periodic event in the community? Nonetheless, the scientific merit of the paper is obvious, the results and conclusions are sounding and up-to-date, and paper should be indexed.

                                                                                                                                

 The paper needs major revisions in its English. The paper is written in a version I dare say is Runglish. There are too many lines in the manuscript that look like a literal translation from Russian of (quite boring) scientific Russian-style. I myself can decipher what the authors mean, since my mother language is also Russian. I am absolutely sure that the current version of the paper will fall out of comprehension for the greatest majority of readers who have no active Russian. To begin with, the title must be changed. No signs, at all. The correct version should be like `` *Evidences of the adaptive mutations in chloroplast genomes of some algae endosymbionts of Baikal sponge*’’.

 Same in the Abstract (Background paragraph): instead of “ *The study of ecosystems of the great lakes is important as observations can be extended to ecosystems of larger scale. The ecological crisis of Lake Baikal needs investigations to discover the molecular mechanisms involved in the crisis. The disease of Baikal sponges is one of the processes resulting in the degradation of the littoral zone of the lake”* there should be something like “ *Monitoring and investigation of the great lakes ecosystem provides a sounding background to forecast the greater scale ecosystem dynamics. Changes in the Baikal lake biota observed nowadays demand deeper investigations of the molecular mechanisms standing behind these former. The endemic Baikal sponge disease may cause a degradation of littoral ecosystem of the lake*”. I am far from the idea that my version is the best, but the original one must be rewritten.

 Unfortunately, there are many more similar problem lines in the manuscript, so very strong revisions in the English are absolutely necessary.

Is the work clearly and accurately presented and does it cite the current literature?

Yes

If applicable, is the statistical analysis and its interpretation appropriate?

Yes

Are all the source data underlying the results available to ensure full reproducibility?

Yes

Is the study design appropriate and is the work technically sound?

Yes

Are the conclusions drawn adequately supported by the results?

Yes

Are sufficient details of methods and analysis provided to allow replication by others?

Yes

Reviewer Expertise:

NA

I confirm that I have read this submission and believe that I have an appropriate level of expertise to confirm that it is of an acceptable scientific standard, however I have significant reservations, as outlined above.

## References

[1] : On the accuracy of statistical procedures in Microsoft Excel 2007. *Computational Statistics & Data Analysis*.2008;52(10) : 10.1016/j.csda.2008.03.004 4570-4578 10.1016/j.csda.2008.03.004

# Response

**Published:** 2020-04-04

**Authors:** Sergey Feranchuk

**Affiliations:** Unaffiliated, Russian Federation

I'm grateful to Prof. Sadovsky for his decision to review this manuscript. I did carefully consider his remarks and prepared a revised version with a respect to his position.

First of all, he was right that crises like the crisis on Baikal could anyway happen in the past. The need to survive in the times of severe crises can be encoded in genome. This idea was introduced to the revised version, as an additional support to the hypothesis about adaptive mutations.

To answer the remark about "closeness" and insufficient robustness of the software, I did several other runs of the assembly. I agree with Prof. Sadovsky about "closeness" and over-complication of some software, and this is why I did choose Inchworm assembler in the initial version of the pipeline, as the most lightweight and straightforward of the available assemblers. In additional runs I tried another assemblers. The correctness of the assembled chloroplast sequence was anyway confirmed, and the fact of verification was pointed out in the second revision.

To answer the remark about "Russian" style of language. This question is in part beyond the scope of the discussion. It is unlikely that me who is Russian will speak the same English as a man from England. But Prof. Sadosvky was right that the meaning of the text in the first edition was unclear in many parts. And in the revised version I put much more attention to a choice of words and grammatic constructions, to use only those words, for which I am certain in their meaning. The text can anyway look unusual to one who know in perfect the context of all words in English, but at least I do my best to make the meaning of the text the most clear.
